# Eruptive superficial porokeratosis in a patient with nephrotic syndrome^☆^

**DOI:** 10.1016/j.abd.2020.08.038

**Published:** 2022-03-20

**Authors:** Masato Ishikawa, Toshiyuki Yamamoto

**Affiliations:** Department of Dermatology, Fukushima Medical University, Fukushima, Japan

## Abstract

A 34-year-old female was referred to our department, complaining of multiple asymptomatic lesions that appeared two weeks previously. The patient had active nephritis with nephrotic syndrome and was treated with immunosuppressive therapies. Physical examination revealed multiple well-circumscribed rounds of flat brownish plaques with slightly elevated borders, some of which were covered by scales. The number of lesions was nine in total. Skin biopsy specimens showed dyskeratotic cells in the thinned epidermis with cornoid lamella, and the absence of a granular cell layer. The development of porokeratosis was considered to be related to immunosuppressive therapy or the activity of nephritis.

Dear Editor,

A 34-year-old female was referred to our department, complaining of multiple asymptomatic lesions that appeared two weeks previously. Physical examination revealed multiple well-circumscribed rounds of flat brownish plaques with slightly elevated borders, some of which were covered by scales (Figure 1, Figure 2). The number of lesions was nine in total: six lesions on the right lower leg, and a solitary lesion on the left lower leg, left thigh and right upper extremity. Skin biopsy specimens showed dyskeratotic cells in the thinned epidermis with cornoid lamella, and the absence of a granular cell layer. Superﬁcial perivascular lymphocytic inﬁltrate in the dermis was also observed (Fig. 3). The lupus band test was negative, and immunostaining for human papillomavirus was also negative. The patient was hospitalized to the Department of Nephrology and Hypertension in our university hospital for nephrotic syndrome and treated with oral prednisolone (25 mg/day), cyclosporine (50 mg/day), and mizoribine (150 mg/day). The patient did not have steroid-induced diabetes. Laboratory data showed abnormal levels of triglyceride (701 mg/dL), total cholesterol (607 mg/dL) and low-density lipoprotein cholesterol (405 mg/dL). Serum immunoglobulin (Ig) G, complements, antinuclear antibodies, anti-DNA antibodies, anti-Sm antibodies, and rheumatoid factor were all within normal ranges. Although the kidney function was normal, proteinuria with hyaline casts was observed, and immunofluorescence examination of renal biopsy revealed granular deposition of IgM and IgG on the basement membrane. Because deposition of complement component 1q was additionally detected, she was initially suspected of lupus nephritis; however, she lacked other symptoms compatible with systemic lupus erythematosus. Topical corticosteroid ointment was applied, but she discontinued the topical therapy.

**Figure 1:**
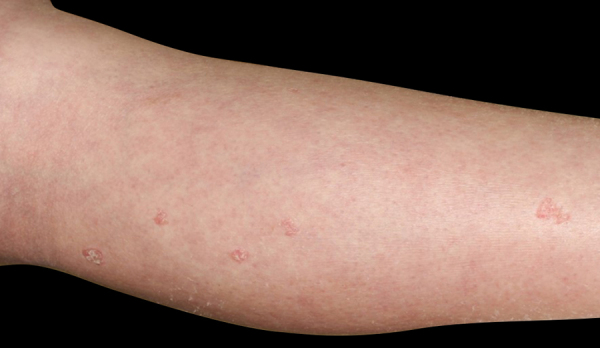
Physical examination revealed multiple reddish keratotic lesions on the right lower extremity.

**Figure 2:**
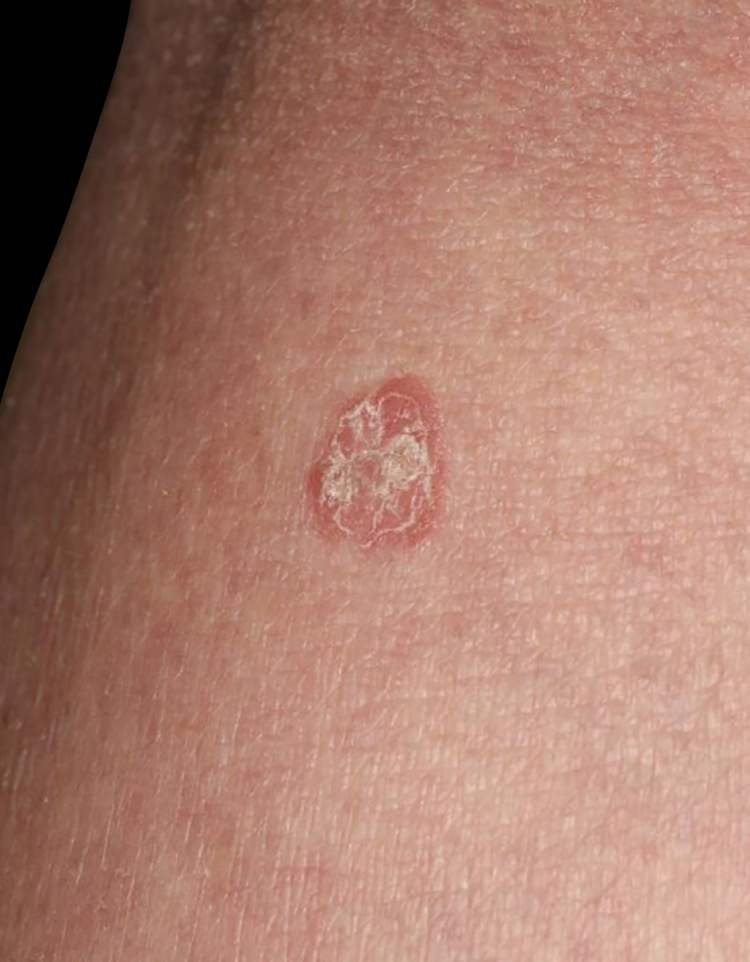
Close-up view showed well-circumscribed, slightly elevated reddish macule with scales.

**Figure 3:**
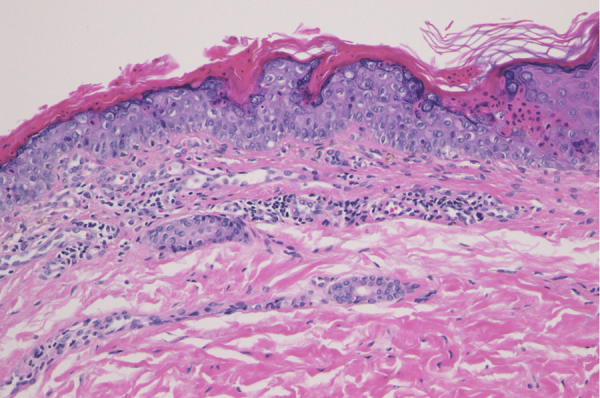
Histological examination showed dyskeratotic cells in the thinned epidermis with cornoid lamella, and absence of granular cell layer. Superﬁcial perivascular lymphocytic inﬁltrate in the dermis was also observed (Hematoxylin & eosin, ×200).

Eruptive porokeratosis is characterized by rapid onset of porokeratosis, which occasionally presents with more than 100 lesions involving multiple regions, in association with paraneoplastic, immunosuppressive, inflammatory, and other conditions.^1^ It is known that porokeratosis develops in association with systemic immunosuppression or under immunosuppressant therapies; however, it is still unclear as to how immunosuppression is associated with the development of porokeratosis.^2^ One possible mechanism is that immunosuppression induces an epidermal keratinocyte population either directly or indirectly.^2^ The abnormal clone of keratinocytes proliferates in a disorderly manner and disturbs the normal growth of the epidermis.^3^ Patients with renal failure rarely develop multiple porokeratosis.^4^, ^5^ Since renal dysfunction can induce various immune regulatory alterations, these cases are suggested to be a new subtype of porokeratosis related to immunosuppression.^5^ In the present case, the patient was initially diagnosed with lupus nephritis. She may develop systemic lupus erythematosus in the future; however, the criteria of lupus nephritis have not been fulfilled as of this moment. In any case, the patient had active nephritis with nephrotic syndrome and was treated with immunosuppressive therapies. The development of porokeratosis was therefore considered to be related to immunosuppressive therapy or the activity of nephritis. Although we are uncertain as to what was the direct trigger for rapid onset of multiple keratosis, given that the patient still showed normal kidney function despite having proteinuria, immunosuppressive therapies may have led to the development of multiple porokeratosis in the present case.

## Financial support

None declared.

## Authors’ contributions

Masato Ishikawa: Designed the study; performed the research and contributed to analysis and interpretation of data; wrote the initial draft of the manuscript; read and approved the final version of the manuscript.

Toshiyuki Yamamoto: Designed the study; assisted in the preparation of the manuscript; read and approved the final version of the manuscript.

## Conflicts of interest

None declared.
